# Activation of proHGF by St14 induces mouse embryonic stem cell differentiation

**DOI:** 10.1007/s13238-016-0282-5

**Published:** 2016-06-18

**Authors:** Xiaoshuang Yan, Yan Xue, Yiye Zhou, Yan Cheng, Shang Yin, Qingwen Ma, Fanyi Zeng

**Affiliations:** 1 Institute of Medical Science, Shanghai Jiao Tong University School of Medicine, Shanghai, 200025 China; 2 Shanghai Institute of Medical Genetics, Shanghai Children’s Hospital, Shanghai Jiao Tong University, Shanghai, 200040 China; 3 Key Lab of Medical Embryo Molecular Biology, Ministry of Health, and Shanghai Lab of Embryo and Reproduction Engineering, Shanghai, 200040 China

**Dear Editor,**

Embryonic stem cells (ESCs) are capable of unlimited self-renewal and differentiation, thus generating remarkable interest in their potential therapeutic use in cell-based tissue engineering and regeneration. Stem cell based therapy has been considered as the most promising therapeutic strategy for a range of debilitating diseases (Reynolds and Lamba, 2014). One key requirement for stem cell differentiation and migration is to overcome existing matrix obstacles, that endowed by multiple mechanisms including tight junctions, as well as physical properties such as the matrix geometry, nanotopography, and stiffness, etc. (Guilak et al., 2009). It was reported that adherent cells showed tensile stress in the cytoskeleton by exerting contractile forces which in turn strengthen the stiffness of the matrix (Ingber, 2004). The strategy cells usually employ to disrupt the matrix obstacle is zymogen cascades mechanism that usually involved at least two proteolytic reactions that can explosively amplify the influence of extracellular signals. During this process, proteolytic activities of serine proteases, one of the most ubiquitous membrane proteases of the protease superfamily, play crucial roles by cleaving the extracellular matrix and enabling the stem cells to translocate to distant target sites (Paule et al., 2015). Facilitating the activation of extracellular signals is essential for the extracellular signaling transduction and fate decision of mESCs, as most growth factors and cytokines are usually synthesized as latent forms that require activation by the protease system located on the membrane of stem cells. Thus, the transmembrane protease system is an indispensable component of the stem cells’ pluripotent program, as it plays pivotal roles in mESCs differentiation. However, the roles of transmembrane protease systems and the impact of interaction between extracellular signals and membrane protease system on pluripotency and differentiation have not been well characterized, leaving significant data gaps that hinder the therapeutic applications of stem cell therapy. Herein we explore the impact of interactions between extracellular signals and the membrane protease system through a specific transmembrane serine protease (St14), and its effect on inducing mESCs differentiation by activating proHGF, one of the substrates of St14. Once cleaved at aa495, the single chain proHGF (inert form) reverts to the active two-chain form of HGF, and mediates a wide range of signaling pathways by binding to the c-Met receptor (Nishida and Hirano, 2003). The activation of proHGF is important in various processes including: placenta development, cancer progression, wound healing, and liver regeneration (Lee et al., 2010; Nakamura et al., 2011).

To detect the expression of St14 in both pluripotent stem cells (PSCs) and MEFs, semi-quantitative PCR and immunoblotting were performed (Fig. 1A and 1B). Using these approaches, we demonstrated that St14 was detected in PSCs, but not in MEFs. This was consistent with the results of quantitative real time PCR (96 × 96 qRT-PCR) (Fig. 1C). Although St14 was differentially expressed in PSCs and MEFs, their differences in the expression pattern was not as extensive as observed in some core pluripotent genes, such as *Oct4* and *Nanog*.

**Figure 1:**
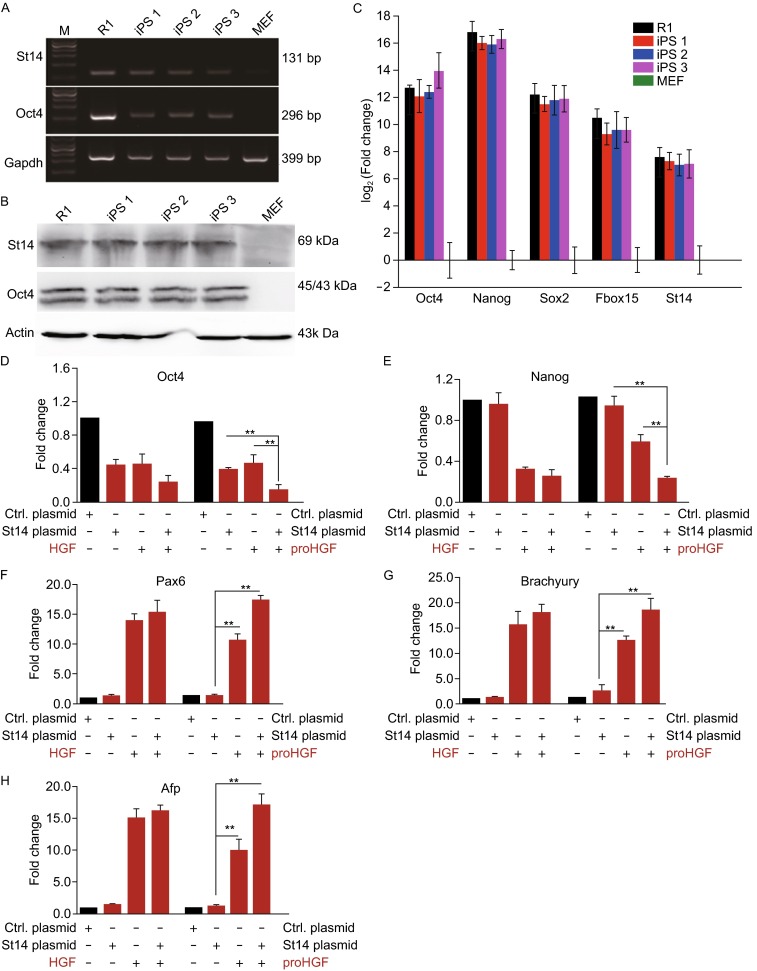
Differential expression of St14 in pluripotent stem cells and MEFs, as well as its activity following proHGF treatment. (A and B) The expression of St14 at the RNA and protein level was detected by semi-quantitative PCR (A) and immunoblotting (B); (C) Log_2_ fold change (stem cell lines vs. MEF) in the pluripotent genes and St14 by 96 × 96 qRT-PCR analysis. Data have been normalized using a reference gene; (D and E) The expression of pluripotent genes in the four treatment groups described in the text; (F–H) The expression of differential genes in the four treatment groups described in the text

In order to better appreciate how St14 functions in PSCs and to examine whether it was involved in regulating pluripotency and differentiation, the expression of select pluripotent and differentiation genes were examined after St14 was up-regulated in mESCs. The results demonstrated that over-expression of *St14* was correlated with decreased expression of Oct4, both at the RNA and protein levels, while Nanog expression was not as significantly affected (Figs. 1D, 1E, and S1). On the other hand, over-expression of *St14* also led to increased expression of the differentiation genes *Tubb3*, *MyoD1*, and *Sox17* (Figs. 1F–H and S2). This suggested that over-expression of St14 may be involved in the differentiation of mESCs.

As up-regulation of St14 appeared to have an important role in mESCs differentiation, we investigated its impact on the activation of its substrate, proHGF, by examining mESCs differentiation after over-expression of *St14* followed by proHGF treatment. The experimental design utilized treatment groups that were designed as: 1) control plasmid, 2) St14 plasmid, 3) proHGF treatment, 4) proHGF treatment + St14 plasmid, and were used to examine the expression of pluripotent and differentiation genes. The HGF treatment served as the control group. The baseline level of proHGF and HGF derived from the medium and/or endogenous proHGF and HGF were firstly detected by an ELISA assay, in order to establish that they would not affect the measurement of mESCs pluripotency and differentiation in our assay (Fig. S3).

The simultaneous over-expression of *St14* and treatment with proHGF (Group 4) resulted in a highly significant decrease in the expression of *Oct4* and *Nanog* than what was observed following either proHGF treatment or overexpression of St14 alone (Group 2 and Group 3) (Fig. 1D and 1E). It was notable that the decrease in the expression of pluripotent genes was comparable to the HGF groups (HGF vs. Group 4 of proHGF treatment groups). Furthermore, over-expression of *St14* increased the expression of differentiation genes on the order of 15-fold in the presence of proHGF, compared to either the St14 (Group 2) and proHGF (Group 3) treatments alone, which were only capable of increasing the expression of the differentiation genes some 5–8 fold, respectively (Figs. 1F–H and S2). Similar results were obtained at the protein level (Data not shown). These results showed that up-regulation of St14 or treatment of proHGF alone in mESCs resulted in slightly increased expression of differentiation genes, but only when St14 was up-regulated can proHGF be maximally effective in inducing significant increases in the expression of the differentiation genes. This suggests that over-expression of *St14* could facilitate the activation of proHGF and promote mESCs differentiation.

 It has previously been extensively reported that the proteolytic activity of St14 was adequately controlled by its cognate kunitz-type inhibitors of serine proteases, HAIs (hepatocyte growth factor activator inhibitors), including HAI-1 and HAI-2. HAI-1 is co-expressed with St14 in various epithelial cells, and can suppress the proteolytic activity of St14 by forming stable inhibitor complexes with St14 (Kojima et al., 2008). Functional studies of this inhibition have been described in various cells, such as tumor cells (Tsai et al., 2014) and placental trophoblasts (Szabo et al., 2009). Here the effect of HAIs down-regulation on mESCs differentiation was examined in the presence of proHGF, together with *HAI-1* and *HAI-2* knock-down (KD) treatments. Both HAI-1 and HAI-2 were found to be highly expressed in mESCs but not in MEFs (Fig. 2A). This was in accordance with the expression of St14. The effect of HAI’s KD on pluripotency and differentiation of mESCs in the presence and absence of proHGF was subsequently examined. Not surprisingly, the results showed that the KD of *HAIs* caused a decrease in the expression of pluripotent genes while increasing the expression of differentiation genes (Fig. 2B). The proHGF treatment produced a more profound decrease in pluripotent genes and increase of differentiation genes than did the other experimental treatments (Figs. 2C, 2D, and S4). This suggested that the KD of *HAIs* had a similar effect as over-expression of *St14* in the presence of proHGF, and both of these processes can enhance the activation of proHGF and induce the up-regulation of differentiation genes.

**Figure 2:**
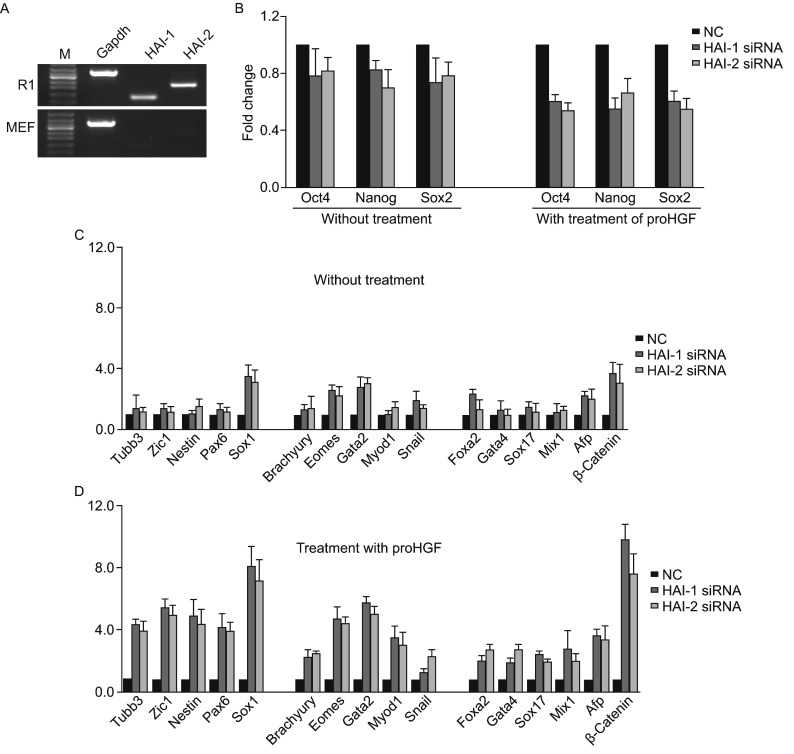
Differential expression of HAIs in R1 and MEF, and their activity associated with proHGF treatment. (A) The expression of HAIs in mESCs and MEFs; (B) The expression of pluripotent genes *Oct4*, *Nanog*, and *Sox2* in HAIs-KD mESCs in the presence and/or absence of proHGF; (C and D) The expression of differentiation genes after KD of HAI-1 and HAI-2 in the absence (C) or presence of proHGF (D)

The abilities of ESCs to differentiate into mature cell types are the prerequisite for their roles in embryonic development and tissue damage repair. Given this developmental plasticity, ESCs represent huge potential value for both regenerative medicine and clinical applications (Whiting et al., 2015). Although stem cells have significant therapeutic potential, they are highly sensitive to various extracellular cues and critically modulated by an elaborate network of cooperation between mESCs and their microenvironment (Brizzi et al., 2012; Chandra and Lee, 2015). As such, their developmental fate could be easily changed by the microenvironment (Liu et al., 2014). Therefore, comprehensive knowledge of the interaction between stem cells and their pluripotent niche was a necessary first step in eventually harnessing their true therapeutic potential. In the present study, we explored the interaction between mESCs and their microenvironment in regulating mESCs pluripotency and differentiation by taking the transmembrane serine protease St14 as an example. It was shown to be differentially expressed in mESCs and MEFs and it mediated mESCs differentiation by activating inert proHGF. Additionally, the proteolytic activity of St14 in mESCs appeared to be correlated with KD of their inhibitor HAI-1 and HAI-2. These results were consistent with the established understanding that during vertebrate development, cellular migration and differentiation were closely related to the pericellular/transmembrane proteases and their cognate inhibitors (Szabo and Bugge, 2011).

It is interesting to note that St14 was differentially expressed between pluripotent stem cells and somatic cells, and its over expression has a positive effect on mESCs differentiation. In addition, the St14 inhibitors HAIs were also highly expressed in mESCs, and the effect of HAIs KD was in line with the over-expression of *St14*, possibly indicating that the activity of St14 in mESCs could be limited by HAIs. Taken together, it appears that St14 most likely functions as a potential switch during mESCs differentiation by activating proHGF. This was also consistent with the observation that over-expression of *St14* alone had no significant impact on mESCs differentiation.

The results of our experiments strongly support our hypothesis that St14 plays a positive role in inducing mESCs differentiation by activating proHGF, and notably, this function could possibly be controlled by manipulating HAIs. In the future it will be interesting to determine the mechanisms underlying just how St14/proHGF induces mESCs differentiation by stable regulation of St14 and the functions of HAIs during mESCs differentiation.

## Electronic supplementary material

Below is the link to the electronic supplementary material.

Supplementary material 1 (PDF 620 kb)
