# Supplementary material for: Activation of proHGF by St14 induces mouse embryonic stem cell differentiation
Source: Protein Cell. 2016 Jun 18;7(8):601–5. doi: 10.1007/s13238-016-0282-5 (PMC4980328; doi:10.1007/s13238-016-0282-5)
Supplement: Supplementary file 1 — Supplementary material 1 (PDF 620 kb) [file 13238_2016_282_MOESM1_ESM.pdf]

Supplementary data

Material and methods

Preparation of feeders and mESCs culture

Feeder cells were prepared using primary MEFs isolated and maintained as previously described, with 10% fetal bovine serum (FBS, PAA Laboratories/GE Healthcare, A15-501) (Takahashi and Yamanaka, 2006). At passage 3 (P3) MEFs were inactivated by mitomycin C (Sigma, M0503) when the cells reach 90% confluence. R1 mouse ES cells, either co-cultured with MEFs or cultured under feeder-free conditions (Sato et al., 2009), were maintained in DMEM containing 15% embryonic stem cell-qualified FBS (PAA Laboratories/GE Healthcare, A15-208) in the presence of 1000U/ml leukemia inhibitory factor (LIF) (Millipo, ESG1107) with supplement of 1mM sodium pyruvate (Gibco, 11360), 0.1mM non-essential amino acid (Gibco, 11140-050), 1% P/S (Sigma, M0503), and 0.1mM beta-mercaptoethanol (Gibco, 21985-023).

Construction of St14 over-expression vector

The St14 coding region sequence (CDS) was cloned into pGM-T vectors after amplification by LA Taq DNA Polymerase (Takara, Cat. RR002A). Primers used for obtaining St14 CDS were listed in Table S1. The cycling conditions were as following: 94 °C for 1min, 32 cycles of 94 °C for 30s, 60°C for 30s and 68°C for 3min. The pGM-T vectors was then cleaved by *Bam*HI (NEB, R0136S) together with *Eco*RI

(NEB, R0101S) to clone the St14 CDS was cloned into pcDNA3.0 (+) plasmids to generate pcDNA3.0 (+)-St14. After transduction into DH5α, the clones were subjected to blue-white selection.

Transfection of target plasmids and HAIs siRNA

pcDNA3.0 (+)-St14 plasmids were transfected into mESCs with 6ug for each well by X-tremeGENE HP DNA transfection reagent (Roche, 06366236001). HAI-1 siRNA and HAI-2 siRNA were transfected by Lipotamine 2000 (Invitrogen, 11668-027). The siRNA used were 5'-AACUGCAACUUGGCGCUAGUdTdT-3' for HAI-1 (Oberst et al., 2005), 5'-GGCAGCCUUUAUAGAUGAAUUdTdT-3' for HAI-2, and 5'-GCTTCATCTACGGCGGGTGCA-3' for the target sequence. Scrambled siRNA was transfected and used as negative control. Twenty-four hours after transfection, HGF and proHGF were added into the medium respectively at a final concentration of 50ng/ml for subsequent experiments.

RNA isolation and cDNA synthesis

Total RNA was extracted by TRIzol reagent according to the manufacturer’s instructions (Life technologies, 15596-018). For cDNA synthesis, 1ug of RNA were used for each reverse transcription reaction with random primers using First Strand cDNA Synthesis Kit (Invitrogen, Cat.C02010A) according to the manufacturer's instructions.

Quantitative real-time RT-PCR

Real-time PCR was performed by FastStart SYBR Green Master Mix (Roche, Cat. 04673484001) using 7900 HT Fast Real Time PCR system (ABI) according to the manufacturer's instructions. The amplification conditions were as the following: 94 °C for 5min, 40 cycles of 94 °C for 15s, 60°C for 15 and 72°C for 40s. The output data was analyzed by comparative Ct method. Primers used for PCR and qRT-PCR were shown in Table S1 and S2 respectively.

Immunoblotting

mESCs were harvested and lysed with RIPA lysis buffer (Santa Cruz, sc-24948). After electrophoresis on 12% SDS-polyacrylamide gel, proteins were transferred onto polyvinlidene difluoride (PVDF) membrane (Millipore, ISEQ00010) by electroblotting. The primary antibodies were incubated with PVDF membrane for 1h after blocking overnight at 4°C using 5% BSA. After three rinses for 5 minutes the PVDF membranes were incubated with secondary antibodies. ECL detection Kit (Millipore, WBKLS0500) was used to detect the protein abundance. The primary antibodies and the concentration used were as the following: anti-Oct3/4 (1:2000 dilution, Santa Cruz, sc-5279), anti-Nanog (1:2000 dilution, Santa Cruz, sc-33760), anti-Actin (1:3000 dilution, Cell Signaling Technology, #4970), anti-St14 (1:2000 dilution, Santa Cruz, sc-48830), anti-Brachyury (1:1500 dilution, Santa Cruz, sc-166962), anti-Afp (1:1500 dilution, Santa Cruz, sc-8108), anti-Pax6 (1:1500 dilution, Santa Cruz, sc-7750). The secondary antibodies were rabbit anti-goat

IgG-HRP (1:3000 dilution, Santa Cruz, sc-2768), goat anti-rabbit IgG-HRP (1:3000 dilution, Santa Cruz, sc-2004), goat anti-mouse IgG-HRP (1:3000 dilution, Santa Cruz, sc-2005).

Identification of proHGF and HGF in mESCs medium by ELISA

The media of different passages of feeders with or without mitomycin treatment were collected and then subjected to enzyme-linked immunosorbent assay (ELISA) using Mouse/Rat HGF Quantikine ELISA Kit (Cat.MHG00, R&D) according to the manufacturer's instructions.

References

Oberst, M.D., Chen, L.Y., Kiyomiya, K., Williams, C.A., Lee, M.S., Johnson, M.D., Dickson, R.B., and Lin, C.Y. (2005). HAI-1 regulates activation and expression of matriptase, a membrane-bound serine protease. Am J Physiol Cell Physiol 289, C462-470.

Sato, H., Amagai, K., Shimizukawa, R., and Tamai, Y. (2009). Stable generation of serum- and feeder-free embryonic stem cell-derived mice with full germline-competency by using a GSK3 specific inhibitor. Genesis 47, 414-422.

Takahashi, K., and Yamanaka, S. (2006). Induction of pluripotent stem cells from mouse embryonic and adult fibroblast cultures by defined factors. Cell 126, 663-676.

Supplementary figures

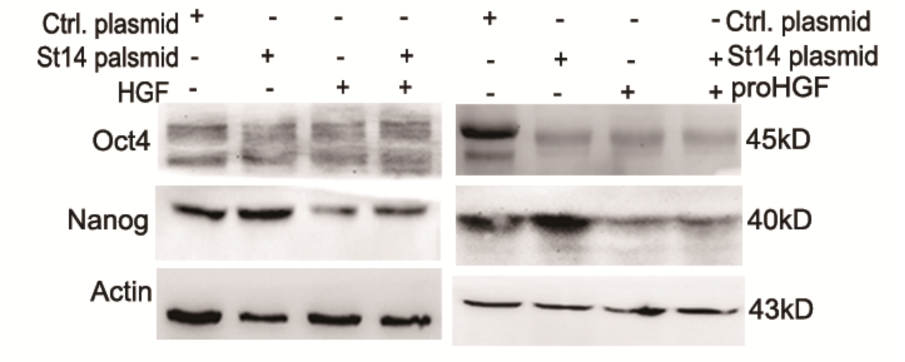

Figure S1. Immunoblotting of *Oct4* and *Nanog* from the four groups mentioned in the text.

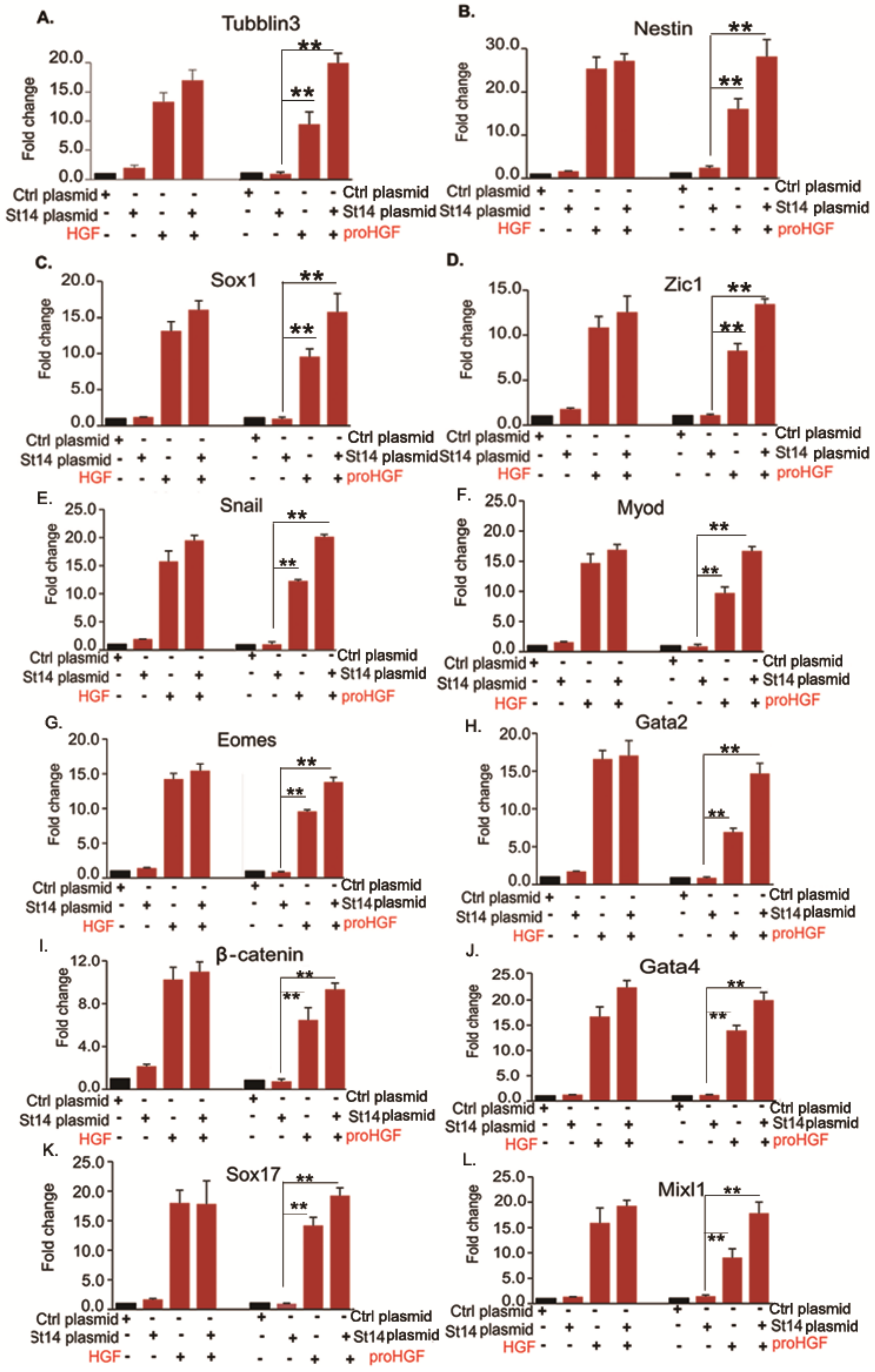

Figure S2. qRT-PCR analysis **of** differentiation genes after over-expression of St14 at the presence of proHGF (left columns) and HGF (right columns). Genes differentiated expressed in the three germ layers were depicted here (ectoderm: A-D; mesoderm: E-H; endoderm: I-L). All experiments were repeated three times. * 0.05>p-value >0.01, **p-value< 0.01.

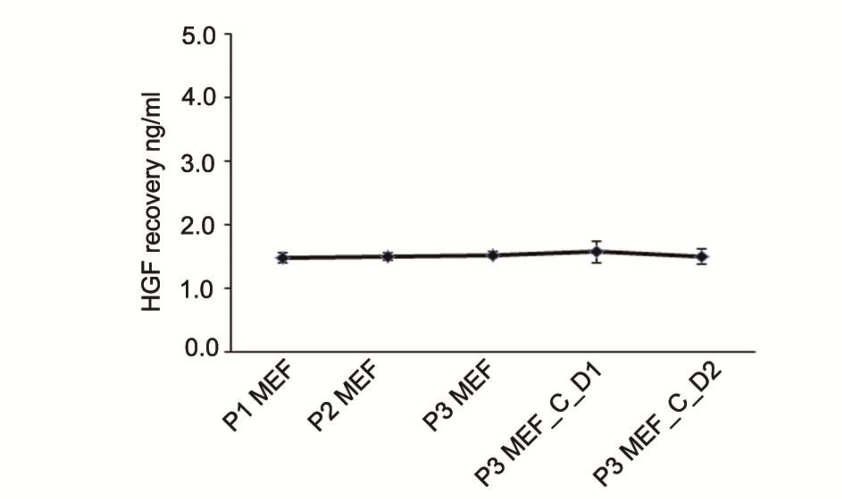

Figure S3. ELISA assay for the detection of baseline level of murine HGF and proHGF derived from the mESCs medium. P1 MEF, P2 MEF, P3 MEF represent MEF medium passages 1, 2, 3 respectively; P3 MEF_C_D1, P3 MEF_C_D2 represent P3 MEFs medium with treatment by cytomycin C for 1 and 2 days, respectively. N=3.

Notes: From this result, the total abundance of proHGF and HGF in the medium was about 1.5ng/ml. This was much less than the Kd of native HGF binding to c-Met, which was reported to be 50-70pM (4.15-5.81ng/ml) (Lokker et al., 1992, EMBO J). This indicated that the basal level of proHGF and HGF in mESCs medium was far less than the threshold necessary for the activation of c-Met in mESCs. Therefore, we conclude the effect of proHGF and HGF derived from the medium could be omitted.

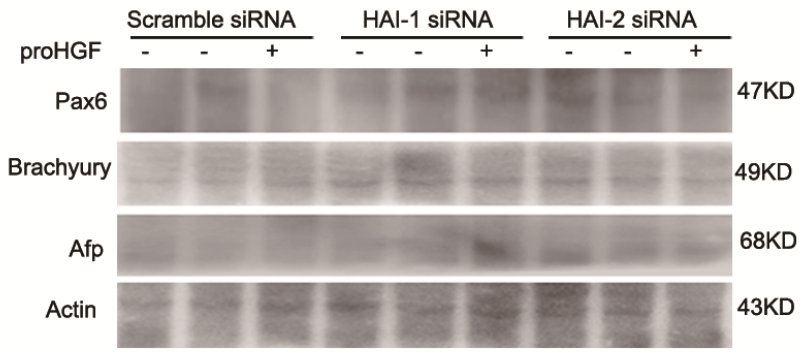

Figure S4. Immunoblotting of differentiation genes after KD of HAIs at the presence (+) and absence (-) of proHGF.

Table S1. Primer sequences used for PCR

Gene	Primer sequences	Product
St14_ for T vector_forward	5'-GGACCGCCAAAACCAT	2568bp
St14_for T vector_reverse	5'-CTATACCCCAGTGTGCTCTT	
Gapdh_forward	5'-GCAAATTCAACGGCACAGTC	399bp
Gapdh_reverse	5'-TCTTCTGGGTGGCAGTGATG	
St14_forward	5'-ATCCTTTACCAAACAGGCTCG	371bp
St14_reverse	5'-AGATGGGGCGCACGAC	
HAI-1_forward	5'-CATCTCTGCCTGCTTCCTCAT	226bp
HAI-1_reverse	5'-TGTGTTGTCTGCCTCGTTCA	
HAI-2_forward	5'-TTTGTTTATGGAGGCTGTGAAGG	100bp
HAI-2_reverse	5'-TGTCATCAGTGGTGTTCTCAGT	

Table S2. Primer sequences used for qPCR

	Gene	Primer sequences	Productct
Pluripotent genes	St14_forward	5'-TCCTACGACTCCAACGACCC	131bp
	St14_reverse	5'-TTGCATCGGCAGTAACGCT	
	Nanog_forward	5'-GCTATCTGGTGAACGCATCTGGAAG	196bp
	Nanog_reverse	5'-AAGTTATGGAGCGGAGCAGCATTC	
	Oct4_forward	5'-TCTTTCCACCAGGCCCCCGGCTC	224bp
	Oct4_reverse	5'-TGCGGGCGGACATGGGGAGATCC	
	Fbx15_forward	ATGGAGGAGTCGGAATTGGAG	114bp
	Fbx15_reverse	GATGGAGGAAGAGCAACGCT	
	Sox2_forward	TAGAGCTAGACTCCGGGCGATGA	297bp
	Sox2_reverse	TTGCCTTAAACAAGACCACGAAA	
Ectoderm	Tubb3_forward	CGGCAACTATGTAGGGGACT	195bp
	Tubb3_reverse	CCAGCACCACTCTGACCAA	
	Nestin_forward	GGACAGGACCAAGAGGAACA	599bp
	Nestin_reverse	TCCCACCTCTGTTGACTTCC	
	Zic1_forward	GCGATCCGAGCACTATGCT	570bp
	Zic1_reverse	GGGTGCGTGTAGGACTTATCG	
	Pax6_forward	5'-AGTACCAGTGTCTACCAGCCAAT	195bp
	Pax6_reverse	5'-GCACGAGTATGAGGAGGTCTGA	
	Sox1_forward	GCGAGGCGATGCCAACT	182bp
	Sox1_reverse	CCCAAAAGAGCGGTAACAACTA	
Mesoderm	Brychuary_forward	GCTCATCGGAACAGCTCTCCAACC	320bp
	Brychuary_reverse	GGAGAACCAGAAGACGAGGACGTG	
	Gata2_forward	GCCGGGAGTGTGTCAACTG	201bp
	Gata2_reverse	AGGTGGTGGTTGTCGTCTGA	
	Eomes_forward	ATCTCCCACGGATTCCCCTA	233bp
	Eomes_reverse	GCTTGTTGGTCACAGGTTGC	
	Myod1_forward	5'-GCTCCAACTGCTCTGATG	273bp
	Myod1_reverse	5'-CCTGTTCTGTGTCGCTTAG	
	Snail_forward	CCGATGAGGACAGTGGCA	365bp
	Snail_reverse	GCAGTGGGAGCAGGAGAAT	
Endoderm	Foxa2_forward	TGCTGGGAGCCGTGAAG	548bp
	Foxa2_reverse	GCTCAGCGTCAGCATCTTGT	
	Gata4_forward	CTCCTACTCCAGCCCCTACC	591bp
	Gata4_reverse	GTGGCATTGCTGGAGTTACC	
	Mixl1_forward	CCGACAGACCATGTACCCAG	146bp
	Mixl1_reverse	GAGGATAAGGGCTGAAATGACT	
	Sox17_forward	TGCGGGATACGCCAGTG	128bp
	Sox17_reverse	CCTCGCCTTTCACCTTTACAT	
	Afp_forward	AGTGCGTGACGGAGAAGAAT	494bp
	Afp_reverse	TGTCTGGAAGCACTCCTCCT	
	β-catenin_forward	CATCTTAAGCCCTCGCTCGG	226bp
	β-catenin_reverse	CAGGTCAGCTTGAGTAGCCAT	
